# Multi-System Adaptation to Confinement During the 180-Day Controlled Ecological Life Support System (CELSS) Experiment

**DOI:** 10.3389/fphys.2019.00575

**Published:** 2019-05-21

**Authors:** Ming Yuan, Marc-Antoine Custaud, Zi Xu, Jingyu Wang, Min Yuan, Carole Tafforin, Loïc Treffel, Philippe Arbeille, Michel Nicolas, Claude Gharib, Guillemette Gauquelin-Koch, Laurent Arnaud, Jean-Christophe Lloret, Yinghui Li, Nastassia Navasiolava

**Affiliations:** ^1^State Key Laboratory of Space Medicine Fundamentals and Application, China Astronaut Research and Training Center, Beijing, China; ^2^Space Institute of Southern China, Shenzhen, China; ^3^Centre de Recherche Clinique, Centre Hospitalier Universitaire d’Angers, Angers, France; ^4^MitoVasc UMR INSERM 1083-CNRS 6015, Université d’Angers, Angers, France; ^5^Research and Study Group in Human and Space Ethology, Ethospace, Toulouse, France; ^6^Institut Toulousain d’Ostéopathie, Toulouse, France; ^7^Centre de Recherche International en Biomécanique, Lagarde, France; ^8^Faculté de Médecine, Unité de Médecine et Physiologie Spatiales, Centre Hospitalier Universitaire Trousseau de Tours, Tours, France; ^9^Laboratory of Psychology Psy-DREPI (EA 7458), Sport Sciences Department, University Bourgogne Franche-Comté, Dijon, France; ^10^Institut NeuroMyogène, Université Claude Bernard Lyon 1, Lyon, France; ^11^Centre National d’Etudes Spatiales, Paris, France

**Keywords:** spaceflight analog, CELSS, Mars mission, chronic stress, adaptation, ethological monitoring, intima-media thickness, muscle tone

## Abstract

Confinement experiments are essential to prepare long-term space exploration. The 180-day Chinese CELSS (Controlled Ecological Life Support System) study is unique in its design, including a closed-loop system and mid-mission simulation of Mars-like day–night cycle of 24 h 40 min for 36 days (days 72–108). Our aim was to study physiological and psychological consequences of this confinement in four healthy volunteers (one female). CELSS platform consisted of six interconnected modules including four greenhouses. Life support systems were controlled automatically. Body composition, fluid compartments, metabolic state, heart, large vessels, endothelial function, and muscle tone were studied using biological, functional, and/or morphological measurements. Behavioral activities were studied by ethological monitoring; psychological state was assessed by questionnaires. Body weight decreased by ∼2 kg mostly due to lean mass loss. Plasma volume and volume-regulating hormones were mostly stable. Carotid intima-media thickness (IMT) increased by 10–15%. Endothelium-dependent vasodilation decreased. Masseter tone increased by 6–14% suggesting stress, whereas paravertebral muscle tone diminished by 10 ± 6%. Behavioral flow reflecting global activity decreased 1.5- to 2-fold after the first month. Psychological questionnaires revealed decrease in hostility and negative emotions but increase in emotional adaptation suggesting boredom and monotony. One subject was clearly different with lower fitness, higher levels of stress and anxiety, and somatic signs as back pain, peak in masseter tone, increased blood cortisol and C-reactive protein. Comparison of CELSS experiment with Mars500 confinement program suggests the need for countermeasures to prevent increased IMT and endothelial deconditioning. Daily activity in greenhouse could act as countermeasure against psycho-physiological deconditioning.

## Introduction

Long-term deep space flights and space colonization in the post-ISS era will be challenged by extreme environment factors including stress, social and environmental confinement in an artificial setting, isolation, sensory deprivation, social monotony, diet modifications with limited available food, and disrupted circadian rhythms (Morphew, 2001). Deep space missions will require high levels of interpersonal compatibility, ability to cope with isolation and group dynamics, capacity for autonomous work, and adaptation to unforeseen challenges. These considerations make crew selection and maintenance of crew performance vitally important. Another constraint is the inability to resupply life-support materials. Such missions will need artificial closed-loop ecosystems to provide crew members with enough breathable air, clean water, food, and a safe environment to live and work – a Controlled Ecological Life Support System (CELSS).

Previous studies suggest that extreme environment factors might induce considerable modifications in physical and mental states, leading to depression, irritability, insomnia, and cognitive impairment (Palinkas, 2002). Preventing medical problems and maintaining health seem more efficient than treatment. Furthermore, early detection of issues, when problems are still mild and resolvable, would allow timely countermeasures, thus diminishing risk to the crew and their mission (Sandal, 2001; Endler, 2004). There is a clear need for easily self-performed techniques to monitor health in space. Obviously, such tools should be validated on ground-based models.

Terrestrial analogs provide standardized high-fidelity experimental conditions (Pagel and Choukèr, 2016). Closed modules allow testing of technologies for autonomous life support systems. They provide preliminary engineering, operational, and biomedical data for an actual mission. Confinement models offer opportunities to study specific aspects of deep space explorations—separately from microgravity and radiation effects of actual spaceflight—to investigate physiological and psychological changes and identify specific ways to address mission-related impairment.

Future manned explorations of the Moon, Mars, and beyond can be better prepared based on the results of high-fidelity simulations. Ground analogs seek to reproduce the elements of anticipated mission. Thus, HUBES (Larina et al., 1997) (“Human Behavior in Extended Spaceflights,” 135-day isolation in a Mir station model, *n* = 3) imitated the Euromir-95 mission, SFINCSS-99 (Custaud et al., 2004) (“Simulation of Flight of International Crew on Space Station,” group 1 with *n* = 4 confined for 240 days in 100 m^3^, groups 2 and 3 with *n* = 4 each confined one after another for 110 days in 200 m^3^) simulated the assembly phase of the ISS program, Mars500 program [550 m^3^ with the Mars-105 pilot study (Gemignani et al., 2014), *n* = 6, and Mars-520 main study (Basner et al., 2014; Gushchin et al., 2014; Jacubowski et al., 2015; Gaffney et al., 2017; Strollo et al., 2018), *n* = 6, module contained an experimental greenhouse but was not designed as a bioregenerative CELSS – with working surface of vegetable greenhouse of only 1.65 m × 0.8 m, food and other consumables were placed inside prior to isolation; water and air were only partially regenerated] modeled spaceflight to Mars. These experiments require considerable work and resources and remain exceptional.

The current project is unique in that it was a closed ecological cycle with minimal outer supply, which was maintained for 6 months. Crew members had to autonomously meet their vital needs of air, water, and food in the relatively spacious CELSS (1,340 m^3^, 370 m^2^) including 888.5 m^3^ of greenhouses. To imitate Mars conditions during the experiment, the crew lived for 36 days on Martian day–night cycle of 24 h and 40 min with realistic interruption of communication with Earth. This long-term simulation was completely organized by China, and all crewmembers were Chinese.

Keeping in mind the need for better understanding and management of risk factors, biomedical investigations were part of the experiment. Our aim was to study the physiological and psychological effects of this 180-day confinement in a CELSS with a 36-day Mars simulation in an integrative manner.

## Materials and Methods

### Volunteers

Four healthy right-handed Chinese volunteers [three males and one female, age 34.2 ± 6.6 years, weight 64.5 ± 6.1 kg, height 169.3 ± 5.1 cm; mean ± standard deviation (SD)] were selected out of 2,110 candidates based on qualification review and physical and psychological checks. Two volunteers were researchers at the Astronaut Center of China (ACC), they became the crew commander and medical doctor/researcher. Two others were recruited at large. Volunteers didn’t practice leisure sport except for one who had table tennis hobby. Pre-confinement VO_2_max consisted 33 ± 4 ml/kg/min and rated as “average” for subjects A and D, “below average” for subject B, and “very poor” for subject C according to sex- and age-specific fitness rating scale (very poor-poor-below average-average-above average-good-excellent).

The study protocol was approved by the ethical committee of the ACC and complied with all guidelines stated in the Declaration of Helsinki. Each subject was informed about the content and schedule of the experiment and signed an informed consent form. The participants were free to withdraw from the study at any time.

### Study Design

**FIGURE 1:**
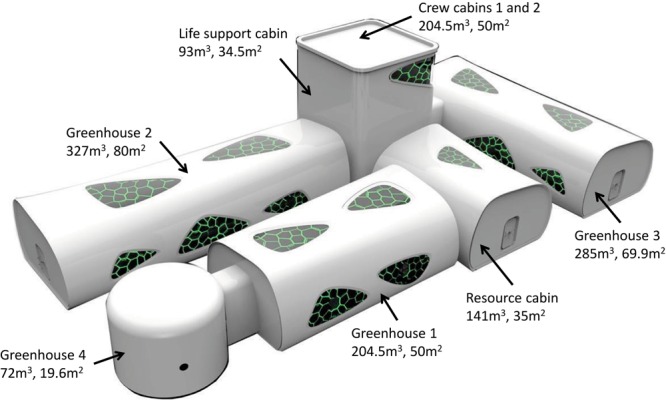
CELSS platform.

**FIGURE 2:**
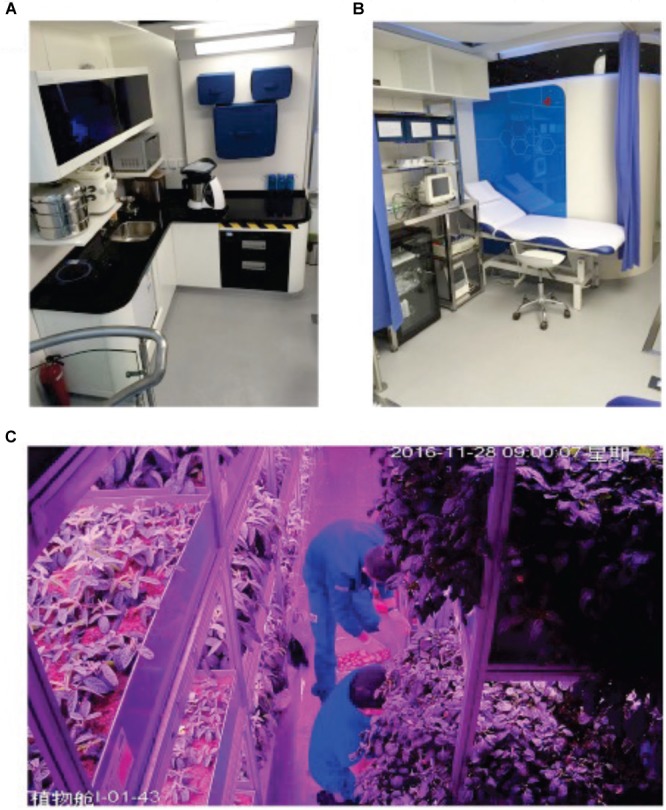
CELSS habitat. **(A)** Kitchen area; **(B)** medical cabin; **(C)** work in greenhouse.

The 4-person-180-day CELSS integrated experiment was organized by the ACC. Confinement in CELSS took place at the Space Institute of Southern China, Shenzhen, China, from June 17, 2016 to December 14, 2016. The CELSS platform contained six interconnected modules and eight compartments including two crew cabins, four greenhouses, a resource cabin with recycling and purification systems to deal with waste (feces, urine, plant debris, waste water, exhaust gas) and to produce CO_2_ for plants, and a life support cabin where food was stored and processed. Crew cabins comprised individual bedrooms, a working area, medical monitoring area, cafeteria, and gym (Figure 1, 2). Life support systems were controlled by automatic feedback networks.

Based on considerations for energy and dietary requirements, as well as multiple taste preferences and health issues, crewmembers cultivated 25 kinds of plants including wheat, potatoes, sweet potatoes, soybeans, peanuts, lettuce, cabbage, edible amaranth, cherry radish, tomatoes and strawberries (for more details on plant species, see Supplementary Table in Dai et al., 2018a). Daily needs in vitamins were planned to be covered by diet (including sea fish) completed by daily dose of multivitamins. However, post-confinement questioning revealed that multivitamin supplementation had not been taken.

During confinement, the subjects were occupied by life support systems maintenance, plant care and household work like cooking and cleaning. Environmental conditions and food and water consumption were monitored. Subjects performed experiments on physiological and psychological effects of confinement. They were able to exercise (treadmill, cycling, core-strength training, dumbbells, resistance bands exercise, tai chi), although it was not mandatory. Besides, VO_2_max testing was performed monthly. Except for tai chi, exercise type and duration were documented. Detailed exercise recommendations were provided to subjects according to their individual fitness as a countermeasure for expected confinement-induced deconditioning. However, these non-mandatory recommendations were not well followed because of workload and insufficient motivation.

Crew worked less on Sunday. Leisure occupations included watching movies, playing cards, reading, etc. The crew celebrated birthdays and holidays. There was no night shift, all volunteers went to sleep and woke up at the same time. For individual hygiene, two subjects used anhydrous baths similar to space station conditions, and two others took showers (Li et al., 2018). Social psychological support included phone and video calls with family and friends every 15 days for 30 min. Otherwise, phone could only be used to discuss the work in cabin with Control room. Crew was not allowed to communicate with outer world via internet. Such limitations in communication aimed to simulate the reality of long-term deep space travel as close as possible. Communication was interrupted during the Martian period, and daily working schedules were emailed. Protocols were set in place in case of medical emergency, but did not need to be implemented. The subjects did not have any medication under confinement except for brief application of topical anti-inflammatory (diclofenac topical gel).

Measurements and samplings were carried out at eight time-points: Baseline data collection prior to confinement (B), six time-points for each month of confinement (M1 to M6), and Recovery data collection post-confinement (R). The subjects were trained to perform measurements and samplings by themselves as usually occurs during isolation studies and space missions. Support manual provided step-by-step descriptions and troubleshooting for each procedure.

CELSS modules were kept under artificial lighting conditions. Crewmembers lived on Mars time from day 72 to day 108, with a day–night cycle of 24 h and 40 min. The Martian period lasted for 36 “Martian” days, which was equal to 37 “Terrestrial” days (53280 min). Otherwise, volunteers lived on Earth time with a 24-h day–night cycle set to 16 h:8 h (16 h 27 min:8 h 13 min for the Martian period). To note, comparable period of 30-day Mars stay was chosen for Mars500 program, accounting for optimal interplanetary travel windows (Tafforin, 2013b; Basner et al., 2014).

### Body Weight, Body Composition, Fluid Compartments, and Plasma Volume Evolution

***Bio-impedance measurements*** with multi-frequency impedance device Hydra ECF/ICF 4200 (Xitron Technologies) were performed as described in Coupe et al. (2013). The subjects were weighed prior to measurements, and ***body weight*** data were used for calculations. We estimated lean and fat mass, as well as total, extracellular, and intracellular water content. Percent changes in plasma volume were calculated from changes in hemoglobin (Hb) and hematocrit (Hct) using the ***Dill and Costill method*** as described in Navasiolava et al. (2010) and De Abreu et al. (2017).

### Blood Studies

Antecubital venous blood samples were collected in the morning before breakfast. Blood samples were analyzed for blood count [red blood cells (RBCs), mean corpuscular hemoglobin (MCH), mean corpuscular volume (MCV), WBCs, Hb, Hct], blood electrolytes [sodium (Na^+^), potassium (K^+^), chloride (Cl^-^), calcium (Ca)], creatinine, fasting glucose, lipid profile, 25-hydroxyvitamin D, hormones [renin, aldosterone, N-terminal prohormone of brain natriuretic peptide (NT-proBNP), adrenocorticotropic hormone (ACTH), cortisol, norepinephrine, prolactin, insulin], enzymes [alanine aminotransferase (ALT), aspartate aminotransferase (AST), alkaline phosphatase (ALP), gamma-glutamyl transferase (GGT), lactate dehydrogenase (LDH), creatine kinase (CK), creatine-kinase muscle/brain (CK-MB)], high-sensitivity C-reactive protein (hs-CRP), soluble endothelial factors [serum CD146, vascular endothelial growth factor (VEGF), E-selectin]. The homeostatic model assessment of insulin resistance (HOMA-IR) index was calculated as fasting insulin concentration (μU/mL) × fasting glucose concentration (mmol/L)/22.5.

### Cardiovascular Studies

#### Cardiospace System

The Cardiospace integrated system was used for monthly cardiovascular studies. It was developed by the CNES and ACC for measurements both on Earth and in space. Of note, Cardiospace equipment was used onboard the Tiangong 2 space lab at the end of 2016, by two crewmembers of the Shenzhou 11 space mission.

Cardiospace tools allow for ECG recording, blood pressure measurement (at brachial level, Holter Tonoport V, Par; continuously at finger level, CNAP OEM, CN-system), ultrasound examinations (Echograph, T3200, Terason), and skin blood flow assessment (Laser Doppler, Periflux 5000, Perimed).

#### Central Hemodynamics and Autonomic Regulation at Rest

Finger blood pressure wave and ECG were recorded continuously for 10 min at rest in a seated position. Beat-to-beat heart rate and blood pressure (systolic, diastolic) were calculated from the signal using specific Cardiospace software. Autonomic cardiac modulation in resting state was estimated via HRV markers – normalized low and high-frequency spectrum power (LF, HF) and spontaneous baroreflex sensitivity as detailed in De Abreu et al. (2017).

#### Cardiac and Macrovascular Morphology and Function

Ultrasound files were recorded by the same subject throughout confinement and analyzed afterward by a specialist, as described in Arbeille et al. (2014). Measurements were performed at the cardiac, carotid, and portal levels. To characterize cardiac morphology and function, left ventricle diastolic volume, stroke volume, cardiac output, aortic velocity and myocardium thickness were estimated. To characterize macrovascular morphology and function, carotid IMT, carotid distensibility, and portal vein diameter were estimated.

#### Endothelial State

Functional microcirculation properties were evaluated at the arm-skin level using laser Doppler flowmetry coupled with iontophoresis, as reported earlier (Navasiolava et al., 2010; Yuan et al., 2015). Iontophoresis of 4% acetylcholine chloride (ACh; 10 s, 0.1 mA anodal current) was applied to assess endothelium-dependent vasodilation. The response was estimated during 20 min following stimulation. Then the probe was continuously warmed to 44°C for 20 min to cause maximal cutaneous vasodilation.

### Muscle Tone, Back Pain, and Spine Mobility

Mechanical characteristics of muscles were determined using a hand-held myotonometer (MyotonPRO; Myoton Ltd.), as described in De Abreu et al. (2017). Feasibility of measurements with the Myoton in actual and simulated microgravity was previously demonstrated in parabolic flight (Schneider et al., 2015), dry immersion (Treffel et al., 2016), and bedrest (Schoenrock et al., 2018). The device was used in multi-scan mode, where one measurement corresponded to the mean of five mechanical taps. Measurements were taken bilaterally on the m. rectus femoris, m. erector spinae at three levels (lumbar, thoracic, cervical), and m. masseter. Results from the right and left sides were averaged. Muscle tone was proxy measured with the “Oscillation frequency” parameter.

A visual analog scale (0–10 with 10 being the worst pain) was used to determine back pain intensity. Spine mobility was determined by measuring hand-to-ground distance during forward flexion (Treffel et al., 2017).

### Behavioral Activity via Ethological Monitoring

Ethological monitoring is based on a quantitative description of spontaneous voco-mimo-posturo-gestual activities during assessment periods. Results of monthly ethological monitoring in CELSS during meals are reported elsewhere (Tafforin et al., 2019). The present paper describes ethological data recorded during the monthly measurements using a Cardiospace system. Monitoring was performed only under confinement, so we have no baseline or recovery data. Each set of Cardiospace measurements lasted approximately 4 h per subject. In total, we analyzed about 90 h of video recording (4 h × 4 subjects × 6 time points) using The Observer XT^®^ 14.0 software (Tafforin, 2017). We measured occurrences of behavioral descriptors (acts per min) on the total analyzed duration per subject. The encoding scheme included ***actions:*** personal actions that subject did individually and interpersonal actions (verbal, visual, body, and object interactions) as manifestation of social interactions; collateral acts (any auto-centered gesture that could be a sign of negative emotions, e.g., touching hair or scratching nose), and facial expressions (indicators of positive emotions and well-being, e.g., smiling); and ***body position:*** sitting, standing, and lying.

Actions (personal vs. interpersonal) and body positions (sitting vs. standing vs. lying) are mutually exclusive behaviors (one can’t sit and stand simultaneously). They are *state events* (duration > 3 s). Besides, *point events* can occur (collateral acts and facial expressions). Behavioral flow as an indicator of global behavioral activity was calculated as a sum of rates (acts/min) for all encoded events (collateral acts + facial expressions + personal actions/interpersonal actions + sitting/standing/lying).

### Psychological Questionnaires

#### Recovery-Stress State

The Recovery-Stress Questionnaire (RESTQ) (Kellmann and Kallus, 2001) was used to assess the balance between psychological stress and recovery states in several dimensions: Individual, Social, Emotional and Physical. The **total stress** score was the mean of scores on seven stress subscales (General Stress, Emotional Stress, Social Stress, Conflicts/Pressure, Fatigue, Lack of Energy, and Somatic Complaints). The **total recovery** score was the mean of scores on five recovery subscales (Success, Social Relaxation, Somatic Relaxation, General Well-Being, and Sleep Quality).

#### Emotions

The Positive Affect – Negative Affect Schedule (PANAS) (Watson et al., 1988) was used to evaluate emotions. Affect refers to whether you feel emotionally in a positive or negative mood. Affective states were categorized into higher-order dimensions of positive and negative emotions (20 items). Positive emotions included the factors active, alert, attentive, determined, enthusiastic, excited, inspired, interested, proud, and strong. Negative emotions included the factors afraid, ashamed, distressed, guilty, hostile, irritated, jittery, nervous, scared, and upset.

#### Adaptation

Adaptation was assessed in emotional, social, physical, and occupational dimensions using the Isolated and Confined Environments Questionnaire (ICE-Q) (Nicolas et al., 2016).

### Data Analysis

Data are presented as individual data and mean ± SD. The small sample (four subjects) with mixed gender (three males and one female) limited the utility of statistical tests, so we only provide descriptive statistics and descriptive analyses.

## Results

### Technological Results

#### Air, Water, and Food Regeneration

In this real-scale simulation, 100% of oxygen and ∼99% of water were regenerated, and ∼70% of food was produced within the module. Air recovery and purification, as well as data on toxic trace gases, are detailed in Dai et al. (2018a,b). Bio-based water recovery is described in Li et al. (2018). In the water cycle, sanitary and other wastewater went to the nutrient solution storage tank for plants, and condensate water from plants was purified with a modified membrane bio-reactor process (Li et al., 2018) and used for drinking and washing. Plant care was performed daily; workload increased during harvest.

#### Environmental Stability Maintaining

Ambient pressure in crew cabins ranged from 81.3 to 104.3 kPa, which corresponds to a (-220 to 1,800)-m altitude. The O_2_ ambient partial pressure ranged from 18.6 to 26.7 kPa, mostly remaining between 19 and 25 kPa. The CO_2_ ambient level ranged from 300 to 700 ppm (Dai et al., 2018a, Supplementary Table). Ambient temperature in crew cabins ranged from 22 to 27°C (mean 23°C). Relative humidity ranged from 40 to 70%. Environmental stability was maintained mainly by automatic control, with additional manual regulation when facility maintenance was performed. To note, maintaining stability in closed-loop ecosystem is a real challenge. Studies with “Biosphere 2” simulation also documented this difficulty (Allen et al., 2003).

#### Food and Water Consumption

Data on diet are presented in Supplementary Table 1. Daily energy intake was ∼2,600 kcal in M1, then slightly decreased to a minimum of 2,000 kcal in M5. Over time, the crew diet included more vegetables from greenhouses and contained fewer fats and more carbohydrates (M1, 35% fats, 50% carbohydrates vs. M5, 14% fats, 65% carbohydrates). Drinking water consumption was unlimited.

Noteworthy, CELSS project was conceived as survival experiment, its main goal was “technological” – to test CELSS, to determine how well food, water, and oxygen can be used, produced and recycled under controlled conditions. Consequently, daily ration was realistically controlled. The availability of food was managed jointly by crew and Control room. “Space” food stored prior to confinement consisted 30% of consumed food. There was no resupply after the onset of confinement. Seventy percent of consumed food was produced in greenhouses, wheat flour and sweet potato were main caloric sources. At the initial period crops covered 55%, and at the period of efficient production – 71% of energy requirements. There were no major food issues during the 6-month period.

#### Exercise and Fitness

**FIGURE 3:**
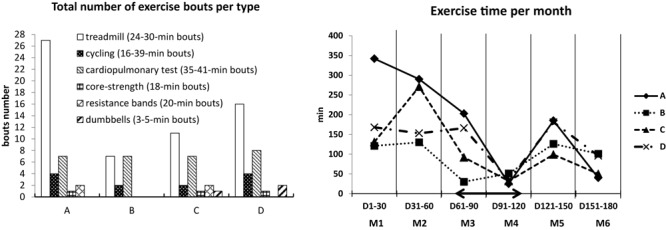
Total number of exercise bouts of different type performed by each subject during 6-month confinement, and global exercise time per month. Range of mean bout durations for individual subjects is reported in legend. Arrow, Mars-like time-shift period (days 72–108).

Total number of exercise bouts of different type performed by each subject during 6-month confinement, as well as global exercise time per month, are shown in Figure 3. Detailed individual data on exercise in CELSS (except for tai chi which was not documented) are reported in Supplementary Table 2. Altogether four subjects performed during 6 months 112 exercise bouts of total duration of 3,117 min (52 h). Treadmill was the most frequent exercise under confinement (15 ± 9 bouts per subject with mean bout duration of 27 min). Subjects exercised less at the second half of confinement, with minimum during Martian period at days 72–108 (time spent per subject on exercise fell from 191 ± 103 min/mo – on M1 to 72 ± 31 min/mo – on M6; at M4 (days 91–120) it consisted 36 ± 11 min/mo).

Monthly VO_2_max test did not reveal substantial modification during confinement (Table 1).

**Table 1:** Clinical and ultrasound data, mean ± SD.

	Ref range	B	M1	M2	M3	M4	M5	M6	R
Body weight, kg	–	64 ± 7	63 ± 5	62 ± 5	61 ± 6	62 ± 5	61 ± 6	61 ± 6	61 ± 6
VO_2_max, ml/kg/min	–	33 ± 4	36 ± 4	33 ± 6	32 ± 2	32 ± 2	31 ± 6	31 ± 5	33 ± 4
DPV, % from baseline	–		+7 ± 6.1	-0.2 ± 8.2	+0 ± 4.5	+3.5 ± 7	+3.2 ± 5.4	+2.4 ± 7.2	+1.8 ± 9.1
HGD, cm	–	19 ± 5	17 ± 3	13 ± 4	15 ± 3	17 ± 3	18 ± 3	18 ± 3	20 ± 5
PVD, mm	7–15	12 ± 0.3		12.4 ± 0.7	11.9 ± 0.2	11.7 ± 0.3	11.6	11.6 ± 0.1	11.2 ± 0.5
LVDV, cm^3^	67–155	114 ± 28		112 ± 43	105 ± 27	102 ± 7	126 ± 25	116 ± 39	110 ± 26
EF, %	> 55	59 ± 4		56 ± 6	65 ± 12	65 ± 4	66 ± 8	72 ± 9	60 ± 4
MT, cm	0.6–1.1	0.89 ± 0.08		0.88 ± 0.04	0.98 ± 0.02	0.95 ± 0.04	0.87 ± 0.05	0.78 ± 0.08	1 ± 0.05
SV, cm^3^	50–100	67 ± 12		61 ± 18	68 ± 19	66 ± 8	83 ± 10	82 ± 16	66 ± 13
CO, L/min	4–8	4.5 ± 0.5		3.8 ± 1.2	4.7 ± 1.2	4.3 ± 1.1	5 ± 0.6	4.8 ± 0.2	4.2 ± 0.8
VAo, m/s	<0.90	0.43 ± 0.04		0.5 ± 0.18	0.57	0.53 ± 0.02	0.53 ± 0.08	0.51 ± 0.06	0.53 ± 0.25

*B, baseline; CO, cardiac output; EF, ejection fraction; DPV, plasma volume change; HGD, hand-to-ground distance; LVDV, left ventricular diastolic volume; M, month; MT, myocardial thickness; PVD, portal vein diameter; SV, systolic volume; VAo, mean aortic velocity; VO_*2*_max, maximal oxygen uptake.*

### Biomedical Results

All four subjects completed confinement without significant medical or psychological issues.

#### Body Weight, Body Composition, Fluid Compartments, and Plasma Volume Evolution

**FIGURE 4:**
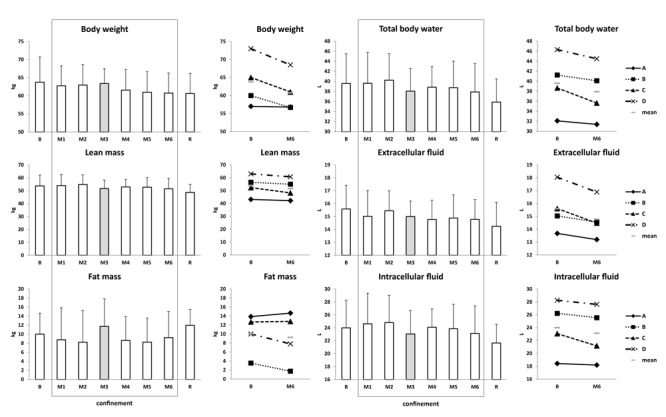
Body composition and fluid compartments. Data are presented as mean ± SD and individual data. Frame, confinement period. Shadowed, measurement point during “Martian” period.

Body weight slightly decreased (64 ± 7 kg at B vs. 61 ± 6 kg at M6) (Table 1). Lean mass tended to decrease (54 ± 8 kg at B vs. 52 ± 8 kg at M6). Fat mass evolution showed large inter-subject variability without clear trends (Figure 4). There was a slight loss in body fluids toward the end of confinement (∼2 L loss at M6 vs. B), similar for extra- and intracellular sectors (Figure 4). Subject C (male) had the highest losses in lean mass and body water (∼15% loss vs. ∼7% average loss for the other three subjects). The estimated change in plasma volume varied within normal limits (Table 1).

#### Blood Studies

**FIGURE 5:**
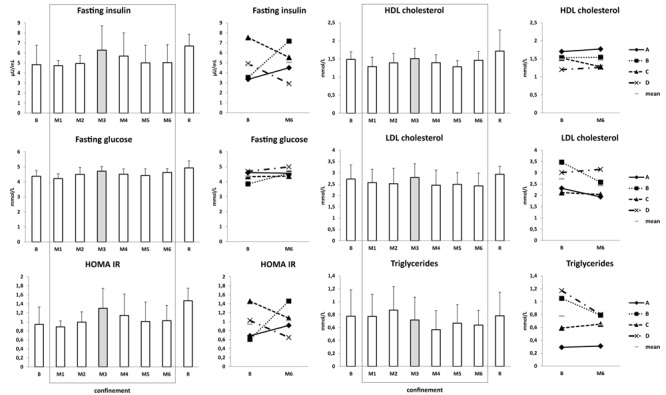
Glucose metabolism and lipid profile. Data are presented as mean ± SD and individual data. Frame, confinement period. Shadowed, measurement point during “Martian” period.

Blood counts remained in the physiological range and were not substantially modified by confinement (Table 2). Electrolytes remained in physiological ranges. Na^+^, Cl^-^, and Ca diminished slightly under confinement in all subjects (Table 2). With regard to ***glucose metabolism***, fasting insulin and HOMA-IR oscillated without clear tendencies, except for subject B who demonstrated moderate increases in M3 and M4. Fasting glucose was not noticeably modified (Figure 5). With regard to ***lipid profile***, high- and low-density lipoprotein cholesterol fractions and triglycerides remained unmodified (Figure 5). 25-hydroxyvitamin D decreased 1.5-fold under confinement (Table 2).

**Table 2:** Blood assessment, mean ± SD.

Variable	Ref range	B	M1	M2	M3	M4	M5	M6	R
Blood count									
RBC, 10^12^ cells/L	4.0–5.5	4.8 ± 0.6	4.6 ± 0.5	5.0 ± 0.6	4.9 ± 0.6	4.8 ± 0.5	4.8 ± 0.5	4.9 ± 0.5	4.8 ± 0.5
MCH, pg	27–31	31.3 ± 1.7	31.6 ± 1.5	30.2 ± 1.6	30.8 ± 1.8	30.2 ± 1.6	31.0 ± 1.6	30.3 ± 1.4	30.7 ± 2.0
MCV, fL	82–95	89 ± 5	89 ± 6	86 ± 5	89 ± 6	88 ± 6	89 ± 6	88 ± 5	89 ± 6
WBC, 10^9^ cells/L	4–10	7.0 ± 1.9	5.7 ± 0.7	5.6 ± 0.7	5.5 ± 0.9	5.7 ± 0.4	5.2 ± 0.6	5.6 ± 1.0	7.3 ± 2.7
Hb, g/L	120–160	150 ± 13	145 ± 10	151 ± 13	149 ± 12	146 ± 12	147 ± 13	147 ± 11	148 ± 9
Hct, %	40–50.8	43 ± 4	41 ± 3	43 ± 4	43 ± 4	43 ± 3	42 ± 3	43 ± 2	43 ± 3
Electrolytes									
Na+, mmol/L	137–147	146 ± 3	140 ± 2	140 ± 2	143 ± 2	142 ± 2	142 ± 3	141 ± 2	142 ± 3
K+, mmol/L	3.5–5.3	4.2 ± 0.6	3.8 ± 0.02	3.9 ± 0.1	3.9 ± 0.1	4.0 ± 0.2	4.1 ± 0.1	4.0 ± 0.1	4.0 ± 0.2
Cl^-^, mmol/L	99–110	104 ± 2.1	101 ± 1.5	102 ± 1.5	102 ± 1.6	102 ± 0.4	106 ± 1.8	105 ± 1.6	104 ± 2.4
Ca, mmol/L	2.1–2.5	2.6 ± 0.1	2.3 ± 0.1	2.3 ± 0.1	2.3 ± 0.1	2.2 ± 0.1	2.3 ± 0.1	2.3 ± 0.1	2.2 ± 0.1
Enzymes									
ALT, U/L	9–50	16 ± 5	11 ± 6	12 ± 6	13 ± 9	11 ± 5	11 ± 7	14 ± 7	24 ± 10
AST, U/L	15–40	18 ± 2	13 ± 4	14 ± 3	12 ± 7	15 ± 5	13 ± 4	16 ± 3	18 ± 7
ALP, U/L	45–125	74 ± 10	61 ± 13	73 ± 12	51 ± 9	61 ± 9	62 ± 11	62 ± 10	62 ± 13
GGT, U/L	10–60	15 ± 4	13 ± 5	14 ± 6	14 ± 6	13 ± 4	13 ± 3	12 ± 3	14 ± 5
LDH, U/L	135–225	251 ± 62	145 ± 17	152 ± 18	152 ± 20	160 ± 18	146 ± 21	143 ± 18	158 ± 22
CK, U/L	38–174	134 ± 56	141 ± 79	99 ± 40	115 ± 42	103 ± 45	127 ± 67	102 ± 32	113 ± 64
CK-MB, U/L	2–25	26 ± 11	11 ± 3	12 ± 3	15 ± 8	13 ± 2	12 ± 2	12 ± 3	12 ± 4
Other									
Creatinine, μmol/L	59–104	79 ± 14	77 ± 13	78 ± 10	74 ± 12	74 ± 12	78 ± 11	76 ± 11	73 ± 14
Prolactin, ng/mL	4–30	17.0 ± 7.2	24.1 ± 5.4	21.7 ± 4.4	12.4 ± 2.7	19.1 ± 8.5	14.2 ± 4.1	18.6 ± 4.8	15.4 ± 5.8
25(OH)D, ng/mL	<12: deficiency; 12–29: suboptimal; 30–100: optimal	22 ± 3	17 ± 4	13 ± 3	16 ± 6	14 ± 4	12 ± 3	13 ± 4	17 ± 5

*ALT, alanine aminotransferase; ALP, alkaline phosphatase; AST, aspartate aminotransferase; B, baseline; Ca, calcium; Cl^-^, chloride; CK, creatine kinase; CK-MB, creatine-kinase muscle/brain; GGT, gamma-glutamyl transferase; Hb, hemoglobin; Hct, hematocrit; K^+^, potassium; LDH, lactate dehydrogenase; M, month; MCH, mean corpuscular hemoglobin; MCV, mean corpuscular volume; Na^+^, sodium; RBC, red blood cells; WBC, white blood cells; 25(OH)D, serum 25-hydroxyvitamin D.*

##### Hormones

**FIGURE 6:**
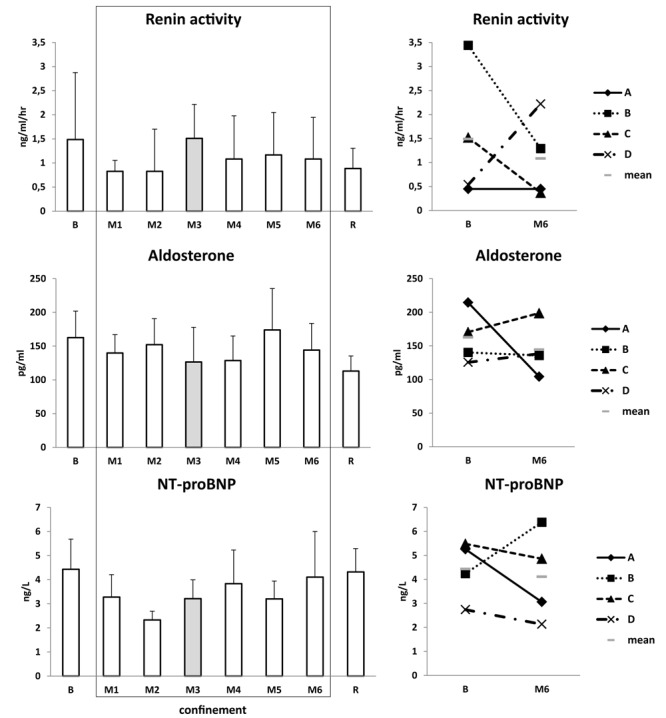
Volume-regulating cardiovascular hormones. Data are presented as mean ± SD and individual data. Frame, confinement period. Shadowed, measurement point during “Martian” period.

With regard to ***volume-regulating cardiovascular hormones***, renin activity tended to decline in M1–M2. Aldosterone oscillated without a clear tendency. NT-proBNP showed slight decline in M2 (Figure 6).

**FIGURE 7:**
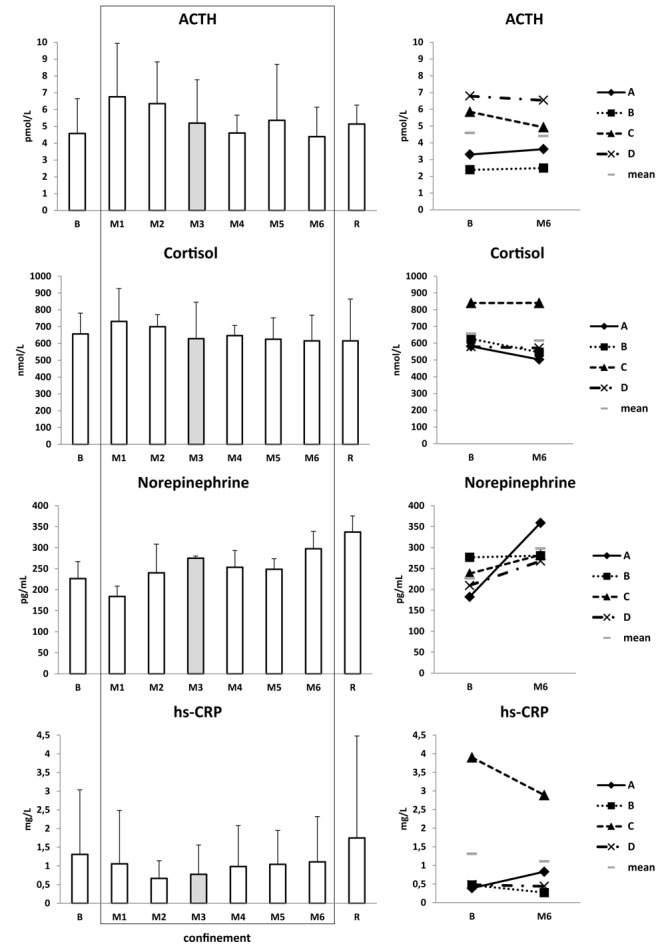
Stress/adaptation hormones and inflammatory state. Data are presented as mean ± SD and individual data. Frame, confinement period. Shadowed, measurement point during “Martian” period.

With regard to ***“stress/adaptation” hormones***, HPA axis (adrenocorticotropin, cortisol) was not substantially modified. At most it showed a modest tendency to increase in M1–M2. Subject C had the highest blood cortisol both at baseline and under confinement. Norepinephrine slightly increased beginning with M3 and remained increased till the end of study (from ∼220 pg/mL at B to ∼340 pg/mL at R) (Figure 7). Prolactin was slightly declined in M3 (Table 2).

##### Inflammatory state

White blood cell number was unmodified (Table 2), and hs-CRP showed a stable low level (Figure 7). Subject C had the highest hs-CRP level before and during confinement. Creatinine and enzymes (ALT, AST, ALP, GGT, LDH, CK, CK-MB) were in normal ranges (Table 2).

### Cardiovascular Studies

#### Central Hemodynamics and Autonomic Regulation at Rest

**FIGURE 8:**
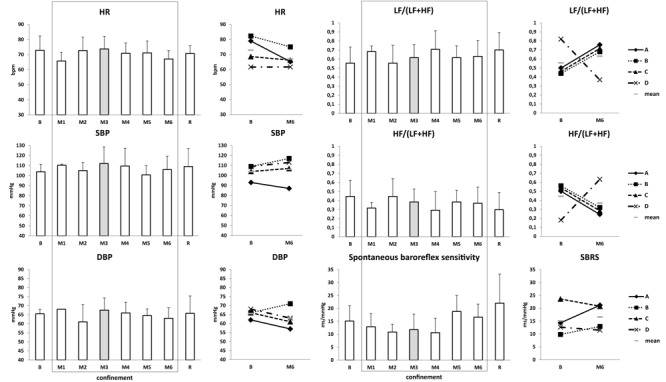
Central hemodynamics and autonomic regulation at rest. Data are presented as mean ± SD and individual data. Frame, confinement period. Shadowed, measurement point during “Martian” period.

Morning heart rate and blood pressure remained normal (Figure 8). HRV markers (LF, HF) and baroreflex sensitivity were not pathologically modified (Figure 8).

#### Cardiac Morphology and Function

Cardiac echography was performed in three subjects. Indicators of cardiac morphology and function (left ventricle diastolic volume, stroke volume, cardiac output, aortic velocity and myocardium thickness) were not pathologically modified (Table 1).

#### Macrovascular Morphology and Function

**FIGURE 9:**
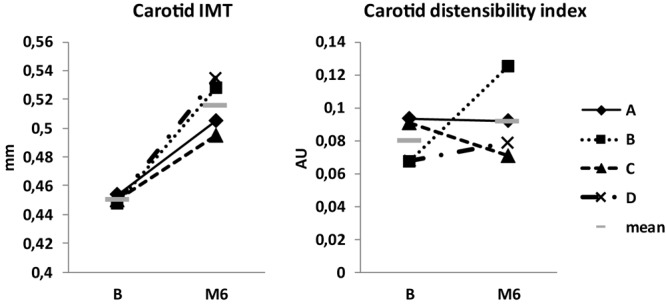
Carotid intima-media thickness (IMT) and distensibility index.

Carotid IMT was increased by 10–15% beginning with M3, in M6 this increase was 14 ± 4% (Figure 9). Carotid distensibility index was not substantially modified (Figure 9). Portal vein diameter tended to decrease slightly (-1 to -6%) (Table 1).

#### Endothelial State

**FIGURE 10:**
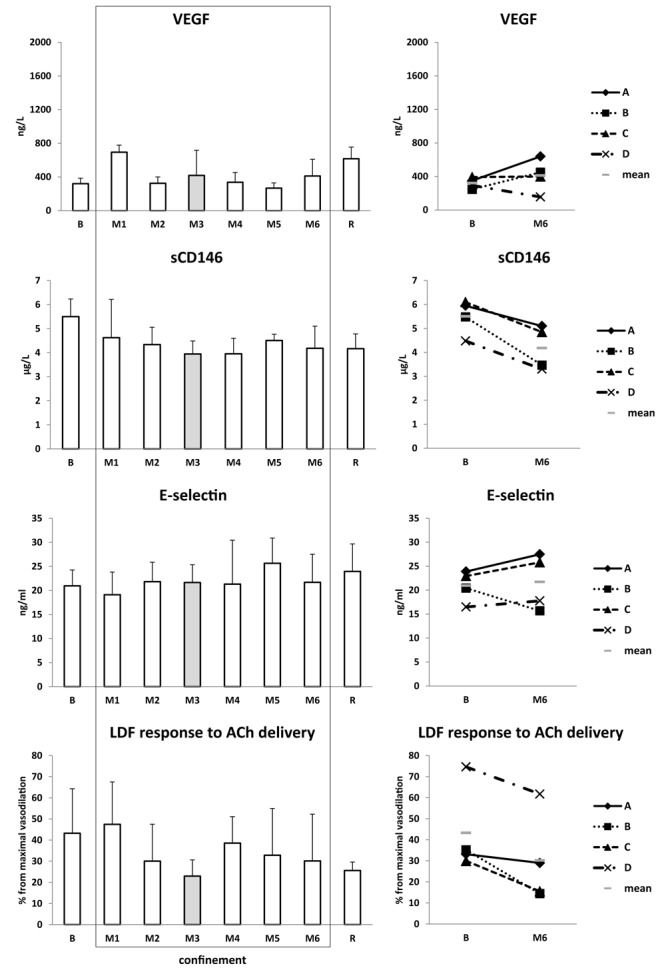
Endothelial state. Data are presented as mean ± SD and individual data. Frame, confinement period. Shadowed, measurement point during “Martian” period.

Vascular endothelial growth factor and E-selectin oscillated without clinical significance. Soluble CD146 slightly decreased. Vasodilation to ACh, expressed in percentage from maximal vasodilation, was decreased by nearly twofold in M3 (43 ± 21% from maximal vasodilation at baseline vs. 23 ± 8% in M3) and slightly decreased at the end of confinement (Figure 10).

### Muscle Tone, Back Pain, and Spine Mobility

**FIGURE 11:**
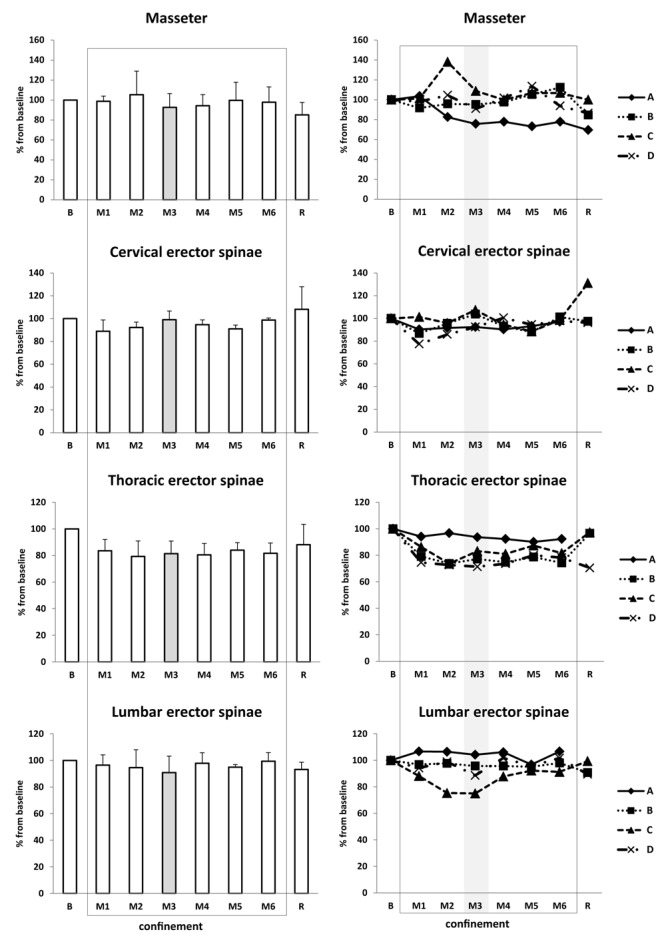
Muscle tone of m. masseter and paravertebral muscles. Data are presented as mean ± SD and individual data. Frame, confinement period. Shadowed, measurement point during “Martian” period.

Masseter tone increased under confinement, with a peak for subject C at M2 (+38%) and for subjects B, C, and D at M5 (+6 to 14%) (Figure 11). Paravertebral muscle tone decreased, with a drop in M1 of 10 ± 6% in average for totality of m. erector spinae (15–20% for cervical, 15–25% for thoracic, and 3–6% for lumbar erector spinae), followed by steady state for M2–M6. Of note, one subject (A) had 6% increase in M1 at lumbar level (Figure 11). For m. rectus femoris, muscle tone was largely unchanged (data not shown).

Three subjects reported periodic back pain with intensity 1 to 5 on a 0-to-10 scale. Pain episodes were related to activities and were relieved by stretching, sleeping, and local analgesics (two subjects applied diclofenac topical gel for 1 week at the beginning of the second month of confinement, in a distance from endothelial assessment at M2 which was at the end of the second month). Mean pain intensity values for the entire confinement period (M1 to M6) were 0.17 for subject A, 0.00 for subject B, 1.67 for subject C, and 1.33 for subject D. Notably, subjects did not report back pain prior to confinement.

Hand-to-ground distance varied without significance (Table 1).

### Behavioral Activity via Ethological Monitoring

**FIGURE 12:**
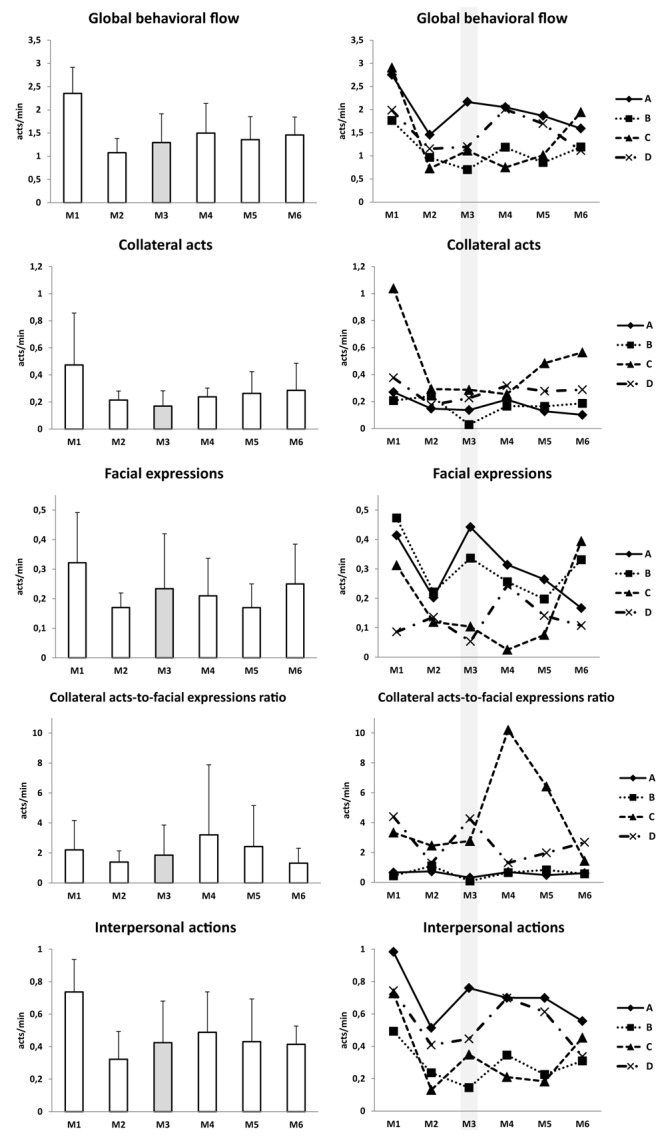
Behavioral data. Data are presented as mean ± SD and individual data. Shadowed, measurement point during “Martian” period.

Data are shown in Figure 12. Behavioral flow reflecting global behavioral activity dropped 1.5- to 2-fold following the first month of confinement, with a minimum in M2 (2.35 ± 0.56 acts/min in M1 vs. 1.08 ± 0.31 acts/min in M2). Similarly, interpersonal actions reflecting social activity were highest in M1 (0.74 ± 0.20), lowest in M2 (0.32 ± 0.17), then mostly stabilized. Occurrence of collateral acts, indicating stress and negative emotions, diminished for the first 3 months (0.47 ± 0.38 in M1 vs. 0.17 ± 0.11 in M3), then gradually increased again for the second half of confinement (0.29 ± 0.20 acts/min in M6). Facial expressions indicating positive emotions and well-being were maximal at the beginning and end of confinement (0.32 ± 0.07 for M1 and 0.25 ± 0.13 for M6) and lowest in M2 (0.17 ± 0.05) and M5 (0.17 ± 0.08).

Subject C had the greatest occurrence of collateral acts throughout confinement, especially in M1. He showed the greatest decrease in global behavior activity compared with M1. The facial expressions rate for subject C was lowest from M2 to M5 and highest on M6. He had the lowest rate of interpersonal actions. The collateral acts-to-facial expressions ratio was highest for subject C (Figure 12).

### Psychological Questionnaires

#### Recovery-Stress State

**FIGURE 13:**
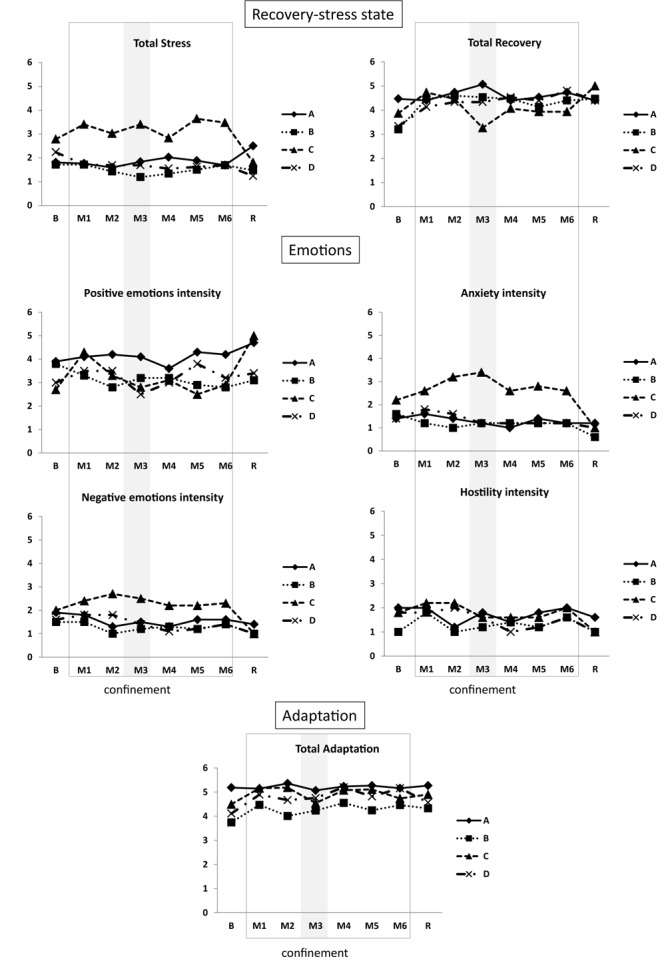
Psychological data. Data are presented as individual data. Frame, confinement period. Shadowed, measurement point during “Martian” period.

Total stress and total recovery integrative variables were stable (Figure 13 and Supplementary Figure 1), and fatigue and conflicts/pressure tended to decrease throughout confinement (Supplementary Figures 2, 3).

#### Emotions

Hostility and negative emotion intensity tended to decrease during the experiment. Positive emotions and anxiety intensity did not change (Figure 13 and Supplementary Figure 1).

#### Adaptation

Total adaptation increased at the first month of isolation and remained elevated (Figure 13 and Supplementary Figure 1). Among the four dimensions (social, physical, professional, and emotional adaptation) only emotional adaptation substantially increased during the experiment (Supplementary Figure 4).

#### Uniqueness of Subject C

Subject C was clearly different from the rest of the group based on his increased level of stress (mean total stress for M1–M6 was 3.4 for subject C vs. 1.6 for the other three subjects), anxiety (2.6 vs. 1.3 for the other subjects), and negative emotions (2.4 vs. 1.4 for the other subjects). At recovery, subject C showed decreases in total stress (and in all subscales relative to stress), anxiety, and negative emotions, and an increase in positive emotions intensity.

## Discussion

Our study aimed to extend the available integrative physiological and psychological data for a simulated flight to Mars within the “4-person-180-day CELSS integrated experiment.” Moreover, our findings about prolonged confinement in closed-loop ecosystem might be valuable for other populations in situations of isolation and confinement (military, submariners, people living in polar regions, residential schoolers, and retirement home residents) and more largely for general adult populations living in isolated conditions, a common social problem in modern western society.

### Main Findings for the Crew as a Whole

Technological results are extensively published and discussed elsewhere (Dai et al., 2018a,b; Li et al., 2018) and suggest the feasibility of technologies used in this CELSS for a long-term mission.

Biomedical results suggest that long-term dwelling in the CELSS does not systematically indicate stress overload and impaired physiological and psychological states. All subjects successfully completed the confinement period. Blood counts, renal function (creatinine), hepatic and cardio-muscular cells state (enzymes) and metabolic state were in normal ranges. Low hs-CRP and stable WBC levels suggested an absence of pronounced inflammatory processes. However, decrease in blood vitamin D suggested insufficient alimentary intake in absence of solar radiation.

Body weight decreased by ∼2 kg mostly due to lean mass loss. Body water slightly diminished, equally for extra- and intracellular sectors. Lean mass loss comparable to water loss suggests muscle loss, probably related to less exercise and activity, and maybe to some caloric decrease. Meanwhile, VO_2_max was not substantially decreased. Plasma volume and volume-regulating cardiovascular hormones were mostly stable. Cardiac morphology and function were not altered. Carotid wall thickness increased 10–15%. Carotid rigidity, estimated by the distensibility index, was not modified. Endothelium-dependent vasodilation decreased by nearly twofold in M3, suggesting endothelial impairment. CD146 slightly decreased. Interestingly, similar decreases in CD146 were noted under 7-day dry immersion and 4-day head-down bedrest (unpublished personal data).

Masseter tone increased, probably in relation with stress (Song et al., 2014). Paravertebral muscle tone diminished. We observed no substantial change in lower limb muscle tone or spine mobility. Two of the subjects experienced moderate back pain, and one had mild back pain.

As for stress/adaptation hormones, norepinephrine increased in the second half of confinement, maybe in relation to the tension of adaptive mechanisms. The HPA axis (ACTH, cortisol) was not substantially activated, suggesting only limited chronic stress. The slight increase for M1-M2 might reflect adaptation to the new environment.

Confined subjects adjusted and adapted their behavior over time, both to the testing conditions and the environmental context within the group. Time records for exercise, already quite modest at the beginning of confinement, further decreased over time. Behavioral flow reflecting global activity diminished after the first month of confinement. Occurrence of collateral acts decreased for the first 3 months, then increased again for the second half of confinement, suggesting increased strain.

Psychological questionnaires revealed adaptive responses throughout the experiment and especially during the first months of confinement, with decreases in hostility, negative emotions, fatigue, and conflict/pressure but higher levels of boredom and monotony reflected by increased emotional adaptation.

### Interrelations Between Systems Evidenced by Findings for a Particular Subject

All subjects showed adaptive responses; however, we observed heterogeneity in adaptation. One male subject (C) was clearly different from the rest of the group based on his increased levels of stress, anxiety, and negative emotions, revealed both subjectively as reported in psychological questionnaires and objectively via behavioral studies. The increased rate of collateral acts for this subject, especially remarkable at the first month, suggests initial stress. Subject C showed the lowest facial expressions rate, greatest collateral acts-to-facial expressions ratio, lowest rate of interpersonal actions, and greatest decrease in global behavior activity, suggesting a shifting of balance between stressful conditions and well-being state toward stress.

He was the only subject to show a peak in masseter tone in M2. He had the highest level of back pain. He also had the highest blood cortisol and hs-CRP levels at baseline, under confinement and in recovery, probably reflecting individual particularities or related to back pain. He had the lowest cardiorespiratory fitness and the highest losses in lean mass and body water.

The increase in facial expressions rate, decreases in all stress subscales, anxiety, and negative emotions, and rise in positive emotions at the end of experiment probably reflect joy, euphoria and well-being underlying subject C’s adaptive state toward completing confinement.

Altogether, we observed unidirectional physiological and psychological changes for this outlier subject, “cross-validating” our integrative findings and suggesting an elevated cost of adaptation and increased stress for subject C. Nevertheless, his recovery scores show that he coped well with the situation. Interestingly, this was a male subject, suggesting that individual differences may be more important than gender differences.

It is known that some individuals are more predisposed to physical or psychological strain, depending on prior experience of coping with similar difficult circumstances (beneficial in critical situations), character and emotional traits, or genetic predisposition (Pagel and Choukèr, 2016). Interestingly, some Mars-520 participants also showed larger shifts in body weight (-21 kg vs. -1 to -11 kg for five other subjects, Strollo et al., 2018) and sleep-wake cycle (Pagel and Choukèr, 2016).

### Carotid IMT Increase and Endothelial Dysfunction

Intima-media thickness increase and endothelial dysfunction are considered cardiovascular risk factors and vascular aging markers. To our knowledge, this is the first study to measure endothelial function in confinement conditions. Endothelial dysfunction occurs after confinement, but apparently at a lower level compared to after head-down bedrest and dry immersion (Navasiolava et al., 2010; Yuan et al., 2015). Decreased daily physical activity is likely responsible for this observation.

A similar increase in carotid IMT was observed in the Mars-520 study (14–28%, Arbeille et al., 2014). This increase could not be attributed to hypovolemia, arterial hypertension, cardiovascular pathology, metabolic alteration (lipid profile, glycemic control), or aging (Arbeille et al., 2014). This suggests that the change is related to the environment. There was no microgravity in the CELSS; the impact on physical activity seemed moderate; and changes in temperature, air, fluid intake, and nutrition were small. However, we should be cautious as it was difficult to quantify these factors. We propose that an inactivity effect suggested by low level of voluntary exercise or nutritional changes (including vitamin D deficit) could underlie this increase in IMT.

### What Was the Effect of “Martian” Day–Night Cycle?

It is known that circadian misalignment by desynchronizing sleep/wake cycle from biological timing system might affect metabolic and hormonal factors (Morris et al., 2012). We are limited in drawing conclusions because this CELSS experiment was not specifically designed to study circadian rhythms. Urine was not collected, and we have only one blood sample and one continuous resting heart rate/blood pressure measurement per point. M3 sampling took place on the 18th day of Martian cycle, and M4 was 12 days after ending Martian cycle. With this in mind, we were unable to assess circadian misalignment. However, no important change in ACTH or cortisol (hormones under clear circadian control, Morris et al., 2012) occurred between M3 and M4. Furthermore, plasma norepinephrine (indicator of sympathetic activity which shows endogenous circadian rhythms) was not notably modified in this time period. Similarly, we did not observe a “step” on M3-M4 for HRV markers (LF, HF) and baroreflex sensitivity as indicators of autonomic cardiac modulation (these reflect endogenous circadian rhythms, i.e., independent of daily changes in behavior or environment; Morris et al., 2012).

Prolactin is primarily regulated by the sleep/wake cycle as opposed to the circadian system (Morris et al., 2012). Its decrease on M3 might indicate shifting sleep/wake cycles. However, questionnaires revealed that only subject A reported a change in sleep quality in M3.

Notably, subject C reported the highest general stress and lowest success, general well-being, and physical adaptation for M3. This subject might be more sensitive to cycle modification.

### Comparison With Mars500

Another study investigating long-term confinement effects during simulated spaceflight to Mars was the Mars500 project with Mars-105 pilot study (Gemignani et al., 2014) and Mars-520 main study (Basner et al., 2014; Jacubowski et al., 2015; Gaffney et al., 2017; Strollo et al., 2018). This joint project of Russian, European and Chinese space agencies, conducted at the Institute of Biomedical Problems in Moscow, aimed to investigate the effects of long-term confinement in a group of six volunteers during a spaceflight simulation to Mars in a 550-m^3^ habitat with four interconnected modules. Mars500 is the project most similar to our CELSS.

#### Mars-105 Results

The effects of mid-term isolation during Mars-105 were limited. Subjective psychological stress level, HPA axis activity (glucocorticoid, catecholamine), and sleep patterns were not altered; there was no acute inflammation or increase in blood cytokines (Gemignani et al., 2014; Strewe et al., 2015; Pagel and Choukèr, 2016). These findings suggest that the Mars-105 protocol did not impose a very strong stress stimulus.

#### Mars-520 Results

All six Mars-520 subjects successfully completed the entire experiment without major health issues. Concerning **glucose metabolism**, insulin and HOMA-IR tended (non-significantly) to increase beginning with M2, and glucose increased beginning with the end of the first year of confinement (M8–M13). Moderate insulin resistance in the second half of Mars-520 was probably partly due to skeletal muscle deconditioning and partly to isolation itself, similar to that observed in people living in polar regions (Strollo et al., 2018). Conversely, in our CELSS, glucose intolerance was not observed. **Lipid profiles** were unchanged in Mars-520 (Strollo et al., 2018), similarly to our CELSS.

With regard to stress/adaptation hormones, **blood cortisol** fluctuated without significance (Wang et al., 2014), similarly to our CELSS findings. It tended to increase only at the end of confinement (day 510). However, **salivary cortisol** increased (Yi et al., 2014; Jacubowski et al., 2015). **Blood norepinephrine** increased toward the second half of confinement (between days 168 and 300), similar to our CELSS observations (Wang et al., 2014).

Considering the inflammatory state, **WBC number** was not significantly changed (Yi et al., 2014), similar to our CELSS results. Also, plasma proinflammatory cytokines (interferon-γ, tumor necrosis factor-α, interleukin-2 and -6) were not modified. However, WBC counts showed a 10% increase in lymphocyte percentage (from ∼43 to ∼54%). Immune responses were also heightened (Yi et al., 2014).

As for body composition, **body weight** progressively decreased mainly due to a **reduction in lean mass** rather than fat mass, as estimated by densitometry (Strollo et al., 2018). This is comparable with our CELSS findings. Body weight loss was more important with Mars-520.

Examining central hemodynamics, the Mars-520 **heart rate** estimated by Holter measurement progressively decreased from ∼77 bpm to ∼66 bpm during the day and tended to decrease from ∼60 to ∼55 bpm at night (Vigo et al., 2013). Conversely, we did not observe major changes in the CELSS, but we performed 10-min continuous measurements and not Holter for an entire day.

As for autonomic regulation, awake HRV showed progressive increase in the amplitude of the HF component (suggesting parasympathetic activation), whereas asleep HRV showed its decrease. This suggested that **parasympathetic circadian rhythms were dampened** (Vigo et al., 2013). The authors proposed it may be due to reduced exposure to daylight related to confinement. In the CELSS, we did not observe circadian misalignment or changes in autonomic regulation.

Considering the macrovascular state, the **increase in carotid IMT** observed in the Mars-520 study (14–28%, Arbeille et al., 2014) was similar to that in the CELSS.

As for **muscular state**, in the Mars-520 impaired performance (loss in muscle force) was observed in the legs despite applied exercise countermeasures, whereas handgrip force was preserved (Belavý et al., 2013; Gaffney et al., 2017). Physical activity, as measured by wrist actigraph and by belt accelerometer, progressively decreased over time (Belavý et al., 2013; Basner et al., 2014).

**Behavioral activity** was estimated in Mars-520 during mealtime. Similar to our CELSS, the Mars-520 crew followed phasic, cyclic, periodic, and punctual behavioral changes in a temporal dynamic as a positive adaptation (Tafforin, 2013a). Behavioral adaptation was not only individual-dependent, like that observed in the CELSS, but also culture-dependent (multi-national crew) (Tafforin, 2013b). Relative occurrence of collateral acts progressively increased (by ∼5% at the end of mission). Relative occurrence of facial expressions diminished in the first half of confinement, then rebounded (Tafforin, 2013a).

As for **recovery-stress state, emotions and adaptation**, there was no increase in subjective stress perception. Responses to the Profiles of Mood State (POMS) questionnaire showed no signs of elevation in total mood disturbance (Basner et al., 2014; Wang et al., 2014; Yi et al., 2014). Visual analog scale ratings indicated low levels of stress, unhappiness, mental fatigue, or tiredness and showed no reliable changes during isolation (Basner et al., 2014; Yi et al., 2014). Similar to our CELSS, Mars-520 was accompanied by emotional adaptation. This adaptation in assessment of affective stimuli (affective pictures from International Affective Picture System, IAPS) was seen as a tendency to assign positive ratings to negative pictures over time. This might be driven by a defensive system, probably activated by increased psychological stress with time (Wang et al., 2014).

#### Limitations of Direct Comparison With Mars-105 and Mars-520 Studies

Both the Mars500 and CELSS facilities were designed as sealed habitats with interconnected modules under artificial light, in absence of natural solar radiation. But direct comparison of the data from these studies is difficult because of major differences in experimental conditions. Mission duration, available space, workload, scenario, nutrition, and operational stress differed. In both Mars500 campaigns, the crew was larger (6 subjects each), international (Mars-520: 3 Russians, 2 West-Europeans, 1 Chinese), and only included males. Furthermore, the Mars500 platform was not designed as a CELSS (although it contained a small experimental greenhouse), so plant care workloads were different (for a typical workday during the Mars-520 simulation, see Supplementary Materials in Yi et al., 2014). Both Mars500 campaigns included mandatory exercise programs. For Mars-520, it was represented by moderate training for ∼30 min daily comparable to that onboard the ISS (Gaffney et al., 2017). However, the Mars500 subjects seem more sedentary than those in the CELSS confinement. Sustained work in greenhouses and life support systems maintenance might better counteract psycho-physiological deconditioning during long-term confinement compared to 0.5 h exercise/day.

Although relatively spacious (550 m^3^ vs. 388 m^3^ of habitable volume in the ISS), the Mars500 platform was substantially smaller than the CELSS platform (1,340 m^3^), mainly due to the absence of “industrial size” greenhouses. Thus, more pronounced changes in Mars500 may be partly explained by the effect of available free space. Restricted space is associated with higher level of stress and inactivity and minimizes opportunity to retreat from the group during tense situations (Pagel and Choukèr, 2016). Caloric intake in Mars500 was more important, pre-defined depending on age and body weight, and consisted of ∼3,000–3,200 kcal/day (for calculation see Strollo et al., 2018), which meant no or limited caloric decrease. The Mars-520 protocol also included 3-step dietary salt reduction from 12 g to 6 g NaCl. Moreover, Mars-520 was three times longer. Previous data suggest that isolation duration may play a major role in deconditioning (Pagel and Choukèr, 2016).

### Chinese Long-Term Confinement in CELSS and an All-Chinese Crew

Historically, access to space was provided by American and Russian space programs. The Shenzhou 5 mission in 2003 made China the third country to independently send humans into space. Now China has successfully launched the Tiangong 1 and 2 space labs, and a Chinese station is planned for the 2020s. The crew in the present study was culturally homogenous, so the participants had a shared language and similar patterns of verbal and non-verbal communication and emotions expressiveness, habits for hygiene and housekeeping, norms for privacy, gender-related behavior, leadership, decision making, coping in relation to conflict, views of experiment goals, and values. This might facilitate compatibility and group cohesion. The monocultural nature of the crew might also explain relative stability of behavior and mood.

On one hand, cultural differences in international ISS crews may be a source of additional difficulties (Ritsher, 2005). On the other hand, Mars500 crewmembers reported that “cross-culture was seen as an advantage” (Urbina and Charles, 2012). International characteristics of the crew might also reinforce cultural influences on behavioral expressions during the dynamic process of adaptation (Tafforin, 2013b). Globally, character traits and personality differences may produce more strain within the team than cultural factors, so crew selection should be based on personality rather than cultural homogeneity.

## Conclusion

In conclusion, this CELSS study simulated a space mission with regard to gender composition, a larger habitat for a planetary destination, and a closed-loop life support system. Isolation and confinement may induce psycho-physiological deconditioning. Systematic investigations will help predict potentially troublesome issues. The results of this CELSS experiment together with Mars500 confinement findings suggest a need for countermeasures to prevent IMT increases and endothelial deconditioning. In long-term missions, psychosocial compatibility of crewmembers (e.g., personality and values) may reduce sources of conflict and improve adaptation to a stressful environment. Sustained daily physical work in greenhouses and life support systems might appear more efficient than exercising for 0.5 h/day to counteract psycho-physiological deconditioning during long-term confinement.

## Data Availability

The datasets generated for this study are available on request to the corresponding author.

## Ethics Statement

The study protocol was approved by the ethical committee of the ACC and complied with all guidelines stated in the Declaration of Helsinki. Each subject was informed about the content and schedule of the experiment and signed an informed consent form.

## Author Contributions

MgY, M-AC, NN, and YL contributed to the conception and design of the study. MgY, M-AC, ZX, JW, CT, LT, PA, and MN contributed to the data acquisition and sample analysis. All authors have contributed to the analysis and interpretation of results, and drafting and revising the manuscript.

## Conflict of Interest Statement

The authors declare that the research was conducted in the absence of any commercial or financial relationships that could be construed as a potential conflict of interest.

## Abbreviations

ACC: Astronaut Center of China
CELSS: Controlled Ecological Life Support System
CNES: French Space Agency (Centre National d’Etudes Spatiales)
CRP: C-reactive protein
DPV: percent changes in plasma volume
ECG: electrocardiography
HOMA-IR: Homeostatic Model Assessment of Insulin Resistance
HPA: hypothalamic–pituitary–adrenal axis
HRV: heart rate variability
IMT: intima-media thickness
ISS: International Space Station
VO_2_max: maximal oxygen uptake
WBCs: white blood cells
