# Supplementary figures and images for: Multi-System Adaptation to Confinement During the 180-Day Controlled Ecological Life Support System (CELSS) Experiment
Source: Front Physiol. 2019 May 21;10:575. doi: 10.3389/fphys.2019.00575 (PMC6536695; doi:10.3389/fphys.2019.00575)

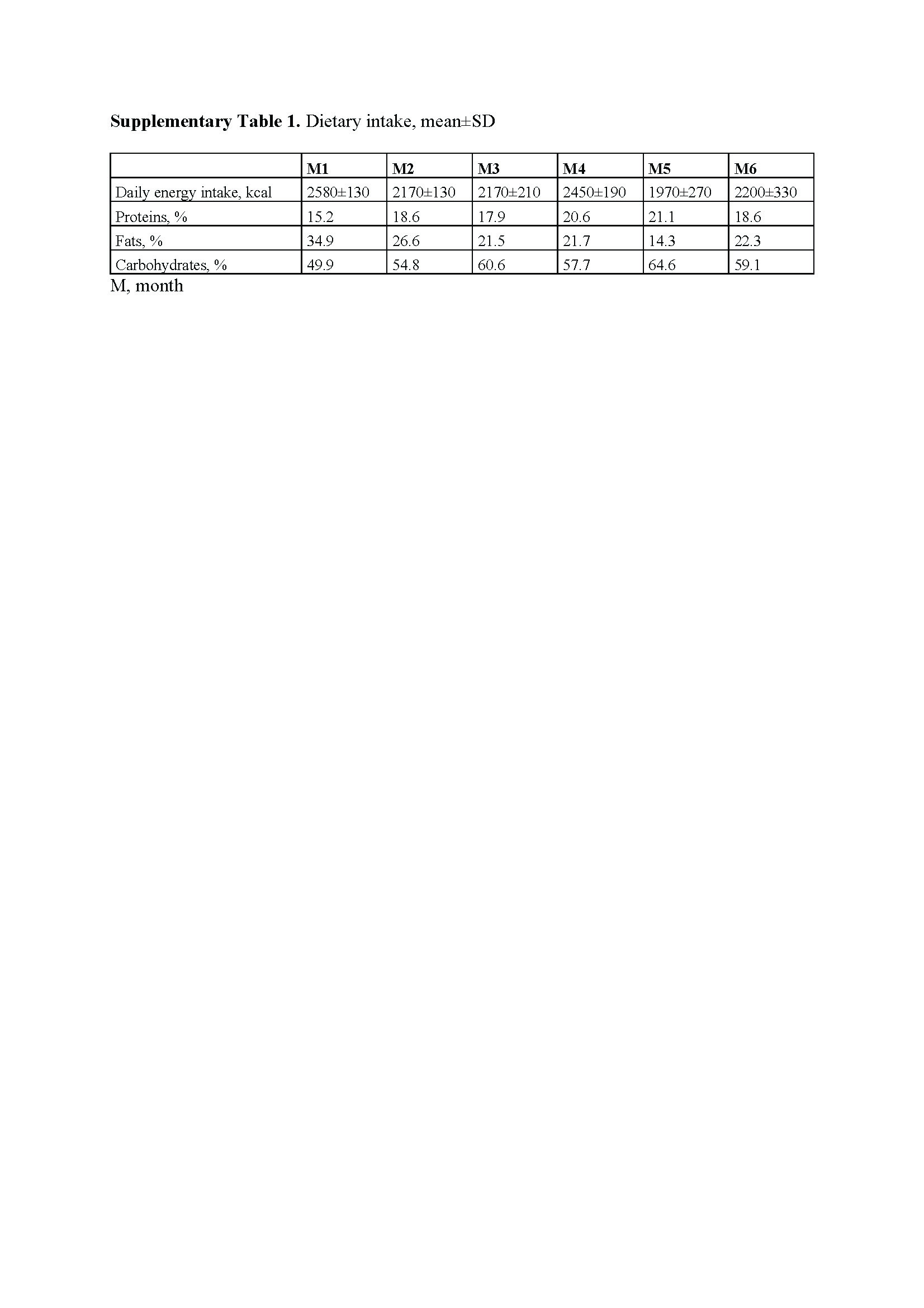

Supplement: Supplementary file 1 [file Data_Sheet_1.zip › Image 1.JPEG]

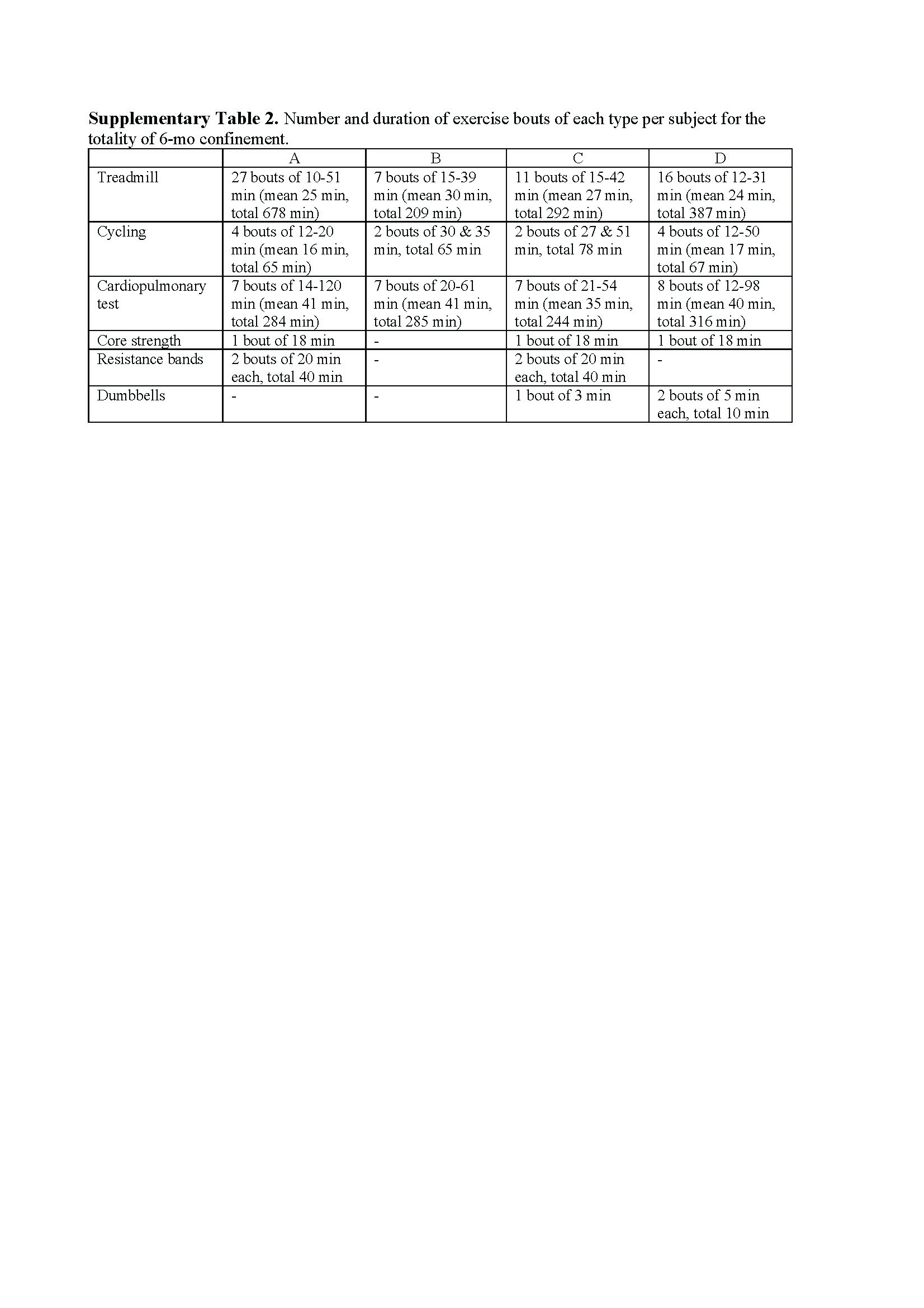

Supplement: Supplementary file 1 [file Data_Sheet_1.zip › Image 2.JPEG]

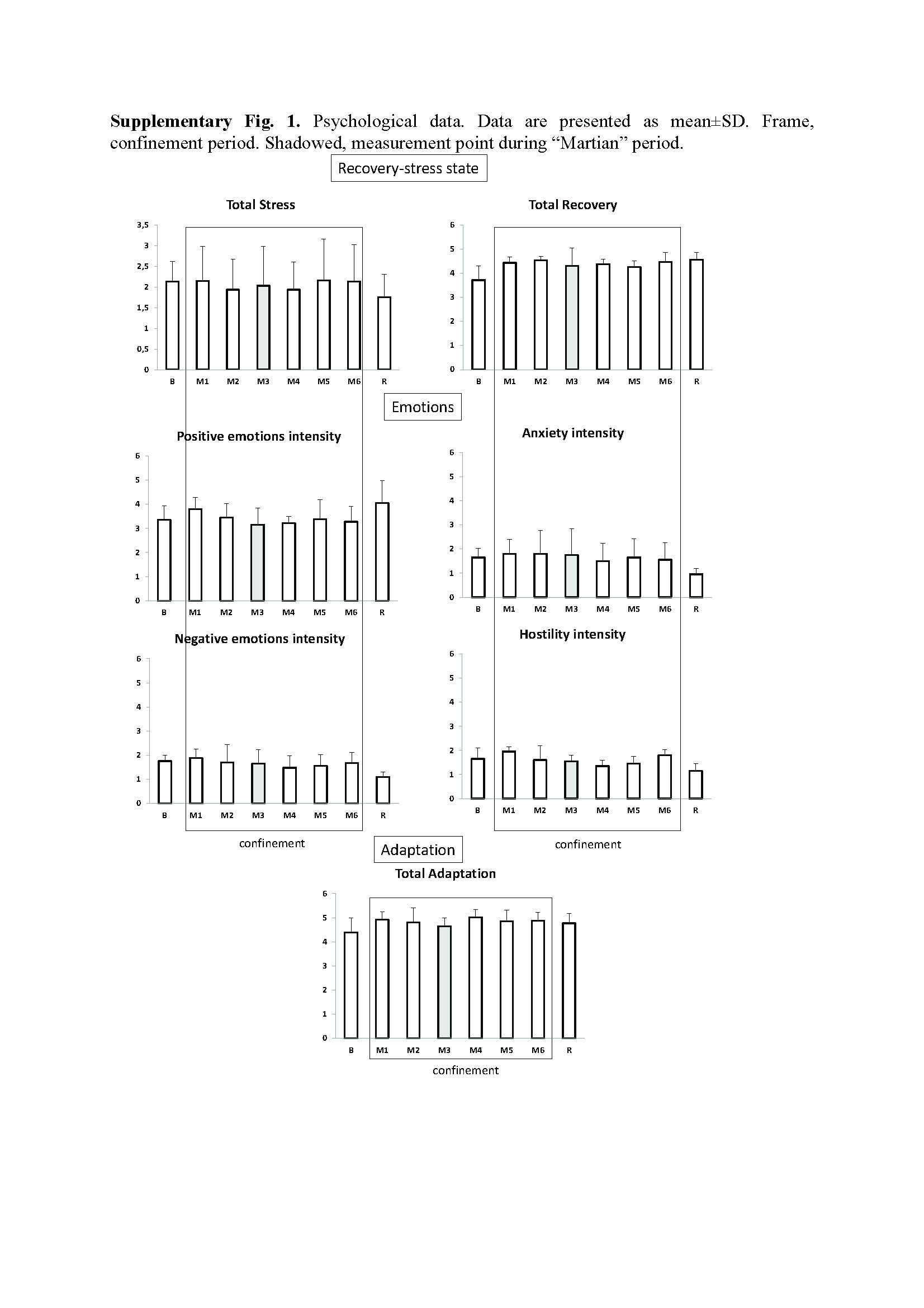

Supplement: Supplementary file 2 [file Image_1.jpeg]

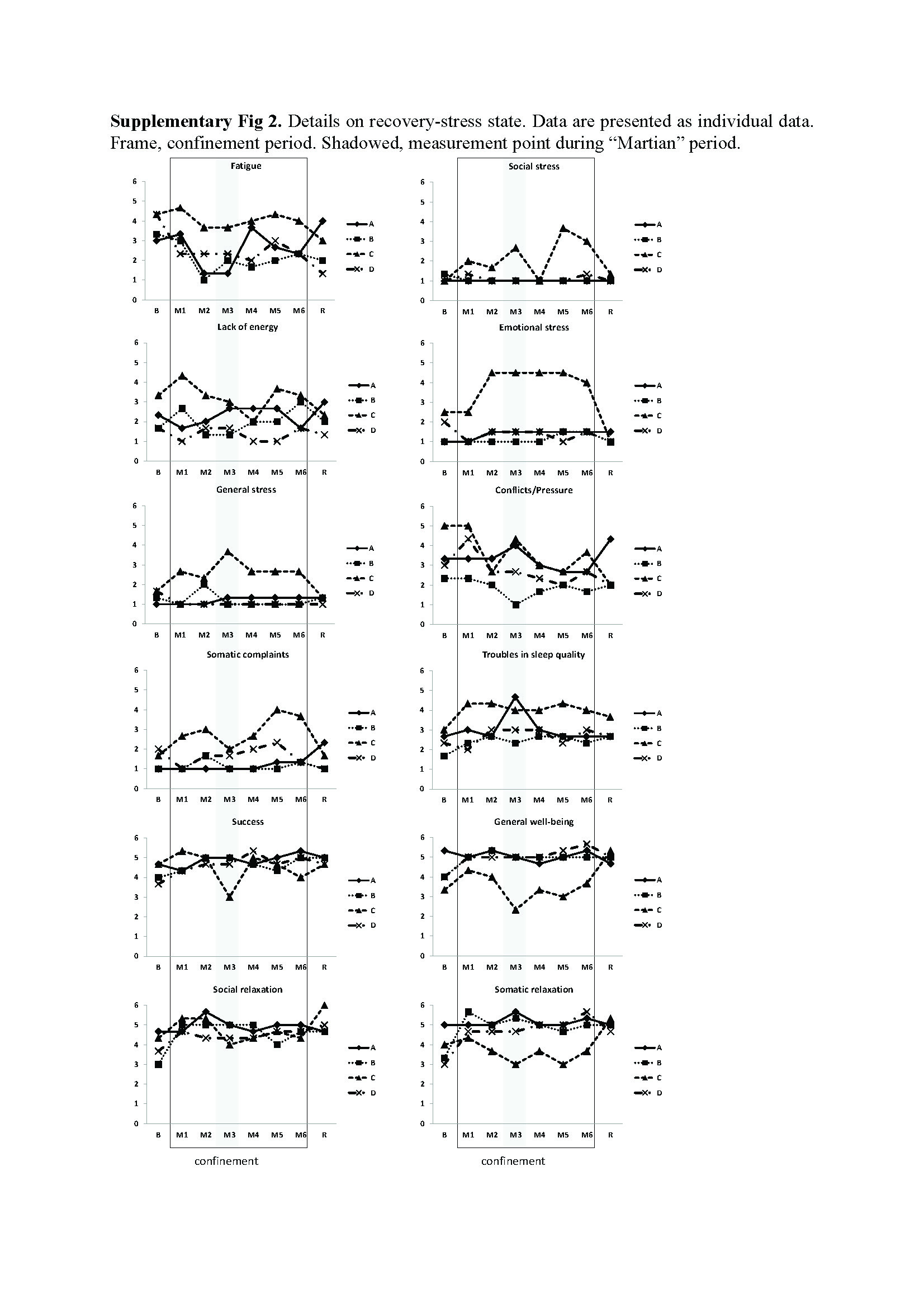

Supplement: Supplementary file 3 [file Image_2.jpeg]

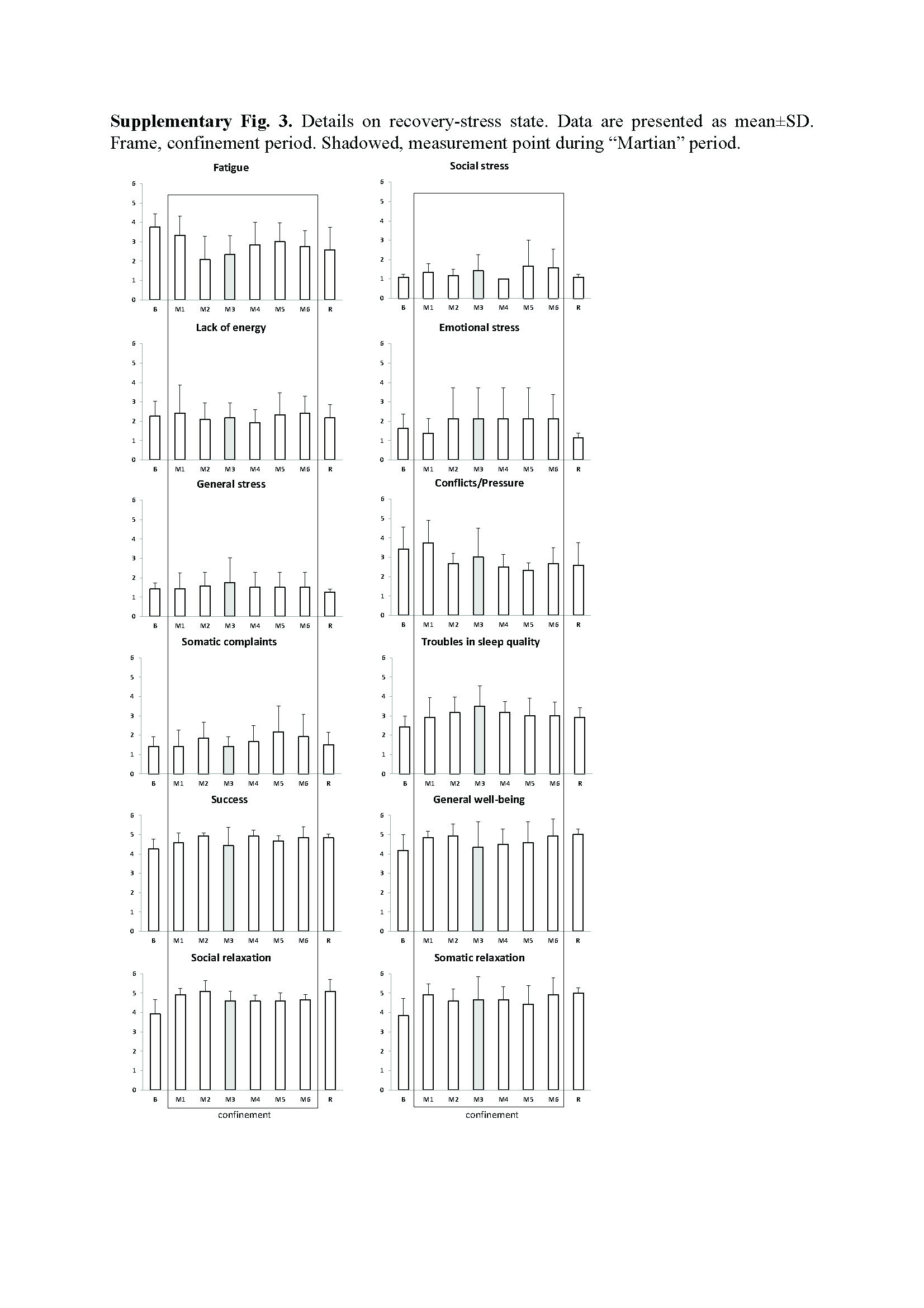

Supplement: Supplementary file 4 [file Image_3.jpeg]

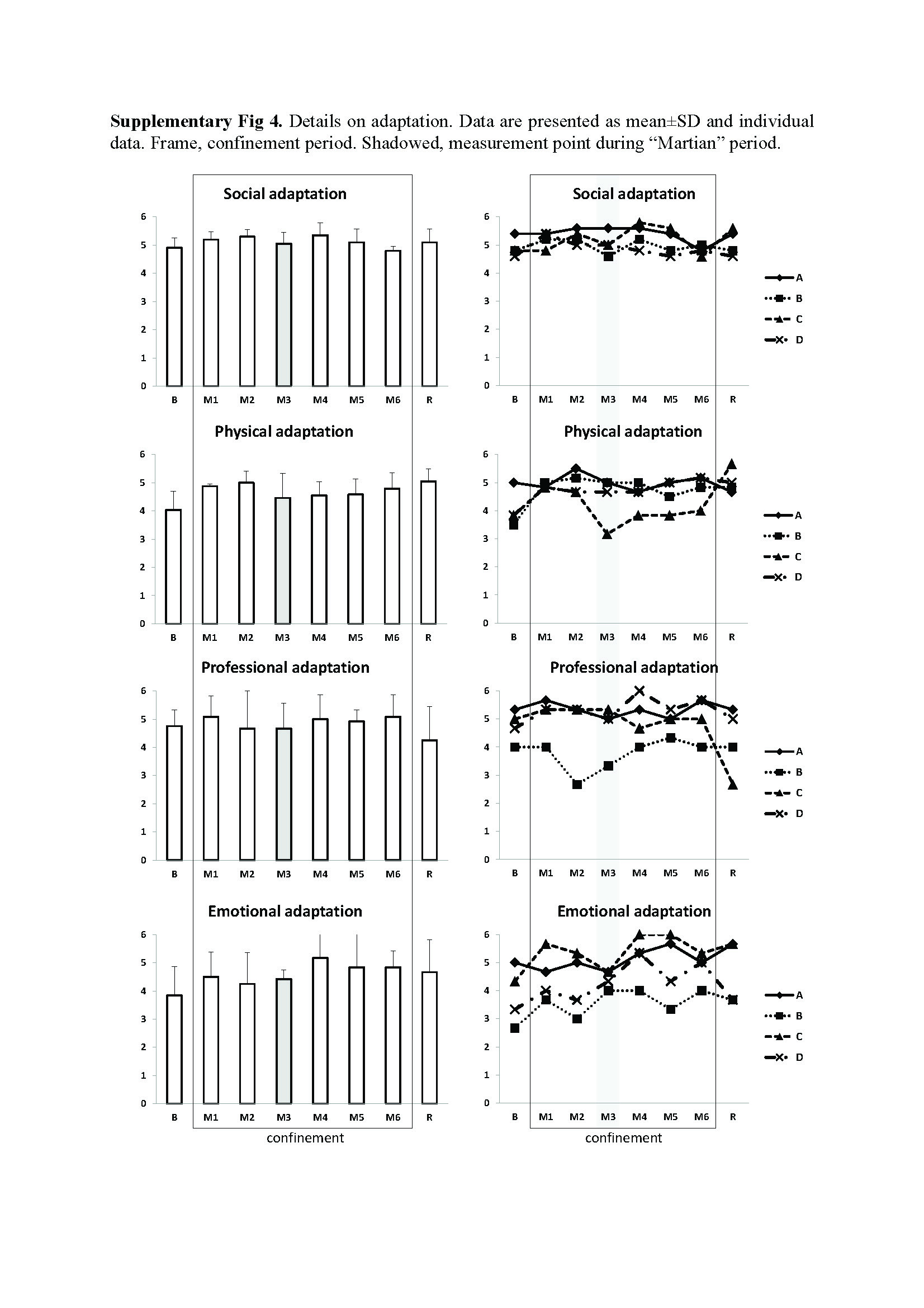

Supplement: Supplementary file 5 [file Image_4.jpeg]
